# Impact of Refractive Errors on the Academic Performance of High School Children of Lahore

**DOI:** 10.3389/fpubh.2022.869294

**Published:** 2022-05-06

**Authors:** Muhammad Zahid Latif, Intzar Hussain, Saira Afzal, Muhammad Asif Naveed, Rahila Nizami, Muhammad Shakil, Abdul Majeed Akhtar, Shabbir Hussain, Syed Amir Gilani

**Affiliations:** ^1^Department of Community Medicine & Medical Education, Azra Naheed Medical College, Superior University, Lahore, Pakistan; ^2^Department of Ophthalmology, Services Institute of Medical Sciences, Lahore, Pakistan; ^3^Department of Community Medicine and Public Health, King Edward Medical University, Lahore, Pakistan; ^4^Department of Hematology, University of Health Sciences, Lahore, Pakistan; ^5^University of Lahore, Lahore, Pakistan; ^6^Department of Biochemistry, King Edward Medical University, Lahore, Pakistan; ^7^University Institute of Public Health, The University of Lahore, Lahore, Pakistan; ^8^Biochemistry Department, University of Health Sciences, Lahore, Pakistan; ^9^Faculty of Allied Health Sciences, The University of Lahore, Lahore, Pakistan

**Keywords:** prevalence, refractive error, refractive corrections, school children, academic score

## Abstract

**Introduction:**

The process of learning begins in childhood and accurate vision can greatly affects a child's learning capacity. It is documented that visual impairment in children can have a significant impact on their performance at school as well as their social interaction and development.

**Objective:**

This research aimed to study the impact of refractive corrections on the academic performance of high school children in Lahore.

**Methodology:**

A total of 2,000 students with equal distribution of gender, public, private school, and locality were included in the study. All students were screened for defective vision. The academic performance before and after corrections was recorded on the prescribed proforma.

**Results:**

The prevalence of refractive error was high among the public high schools 244 (59.2%) as compared to the private schools 168 (40.8%). The area-based prevalence was higher among the students in urban settings 255 (62%) while in rural it was 157 (38%). It was found that in the public sector, the average score of academic results before the intervention was 56.39 ± 13.24 which was increased to 60.27 ± 14.94 after the intervention while in the private sector, before the intervention, the average score was 63.53 ± 17.50 which was improved to 67.12 ± 18.48. It was found to be statistically significant at *p*-value < 0.05.

**Conclusion:**

A significant impact was observed in the average academic scores of the results after refractive corrections.

## Introduction

Refractive errors are considered an important public health problem affecting people all over the world. These errors are classified into three types: myopia, hypermetropia, and astigmatism (1). Generally, the reports about the link between factors associated with education and refractive errors among the children of high school levels imposed major consequences on public health.

According to the estimates of the World Health Organization (WHO), 285 million people are visually impaired including 246 million having low vision and 39 million blind (2). Diverse community-based studies of uncorrected refractive errors (URE) in different countries conclude a prevalence of 22.3% in China, 17.3% in Singapore, 17.1% in Malaysia, 15.8% in Chile, 10.2% in Australia, 10.2% in Bangladesh, and 6% in the United States, respectively (3). However, in contrast to the above-mentioned prevalence, a school-based study among the students aged 5–16 years from Rawalpindi reported a prevalence of 3.35% (4). Research studies have also revealed that the most common reason for the visit to an ophthalmologist or eye care professional is related to refractive errors (5).

Refractive errors can have many issues including educational loss, economic issues, low productivity, and impaired quality of life (6). The available literature identifies that a significant number of Pakistani school children are suffering from refractive errors (7). A relevant study concluded that every 5th Pakistani high school student is having a visual disability in the form of refractive errors, which reflects a serious threat to the individual and societal academic achievements (8). A study conducted in Bengaluru, India, showed a prevalence of 10% whereas the prevalence found in different relevant studies from Pakistan was 19.8% in Lahore, 20.43% in Kohat, 17.24% in Southern Punjab, and 21.7% in Lahore, respectively (8–10). Another research among the students of a religious school (Madrassa) at Haripur showed a high prevalence of 41% in the refractive errors (11). Moreover, a significant impact of the visual acuity on the academic performance of school students has also been shown in a study from Nigeria (12). For instance, the occurrence of the refractive error in the educational system fluctuates according to the ethnicity and area. It is a serious matter to carry out an updated study on school-level Pakistani students to explore the association of the refractive errors with the academic performance.

Lahore is the second-largest city of Pakistan, provincial headquarter, and a historic cultural center of South Asia. The city is known as the educational capital of Pakistan and includes public and private education institutions of international standards. The public sector is governed by the provincial government with minor financial burdens on the learner while the private schools are led by organizations with variable fee structures. The reasonable socioeconomic status of the parents, availability of financial resources, and comparatively perceived better level of awareness regarding child health were the major reasons to include the private sector high school students along with the students of public sector high schools in this research.

This research aims to study the impact of refractive corrections on the academic performance of high school children in Lahore.

## Methods

Before the start of the study, ethical approval was granted by the Institutional Research Board of the University of Lahore vide letter reference number IRB-UOL-FAHS/00248A. The Helsinki Declaration (modified in 2013) was followed due to the inclusion of human subjects. Also, the permission of the district administration was obtained from the Education Department to recruit the children of the high schools. Written informed consent was obtained from each participant or guardian or teacher to execute the process.

### Study Population

**Figure 1:**
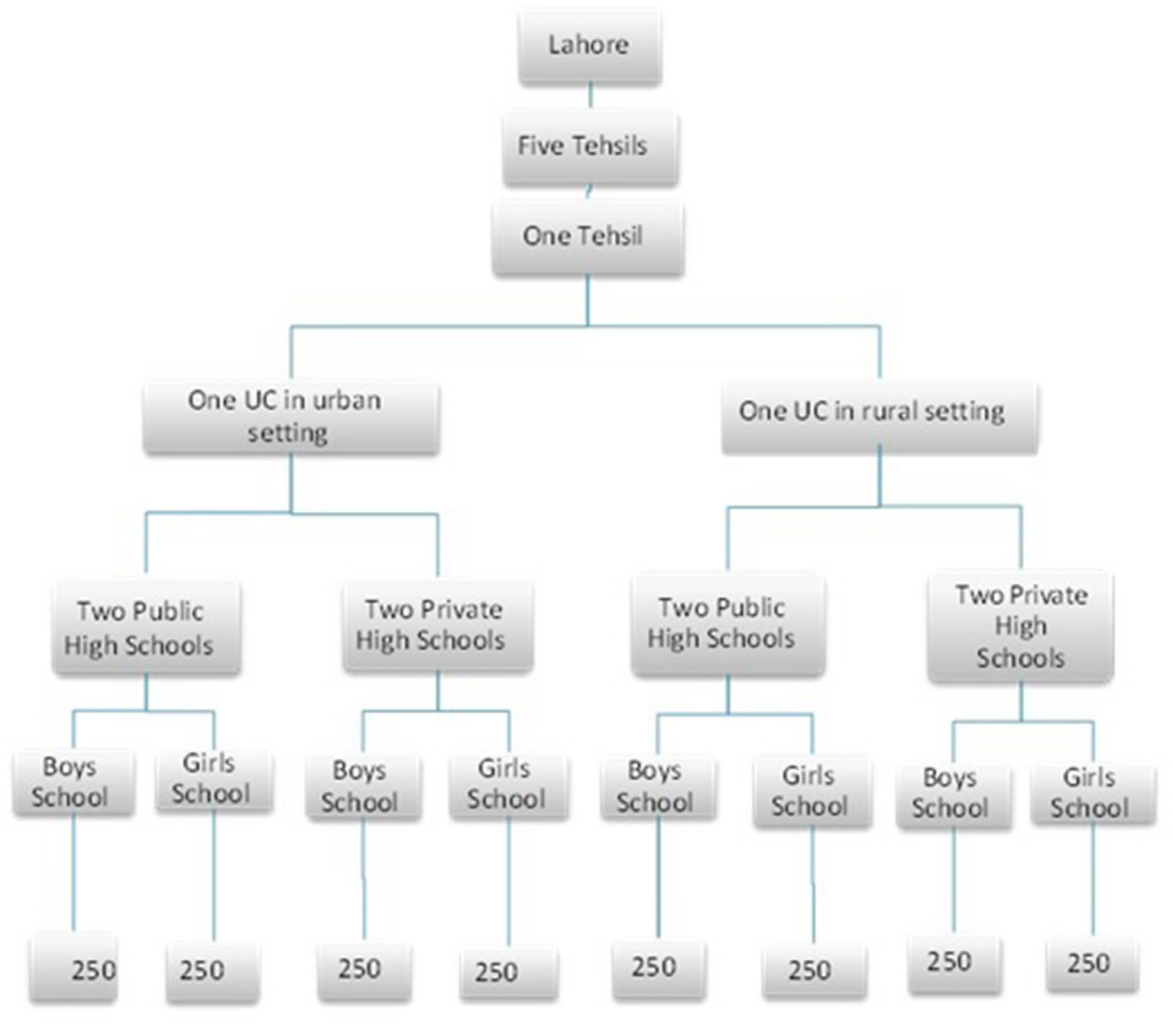
Flow diagram showing the sampling of study subjects.

The study was carried out on 2,000 children in high schools in Lahore. The sample size was calculated by the Open Epi Tool kit (13). Multistage random sampling technique opted for the recruitment of the subjects. Out of the five tehsils (a local unit of administrative division) of Lahore, one tehsil was selected randomly followed by the random sampling of one union council (UC) from unban and one UC from the rural setting of the selected tehsil. Later, a list of the public and private boys' and girls' high schools was obtained and one school from each category was randomly selected as presented in Figure 1. Children of high school levels (from class 6th to class 10 th) with both genders (boys and girls), from the public, private sector, and rural, urban settings were included in the study. The children with ophthalmic infections, visual acuity <6/6, and coexisting organic defects were excluded from this study.

### Data Collection Process

**Figure 2:**
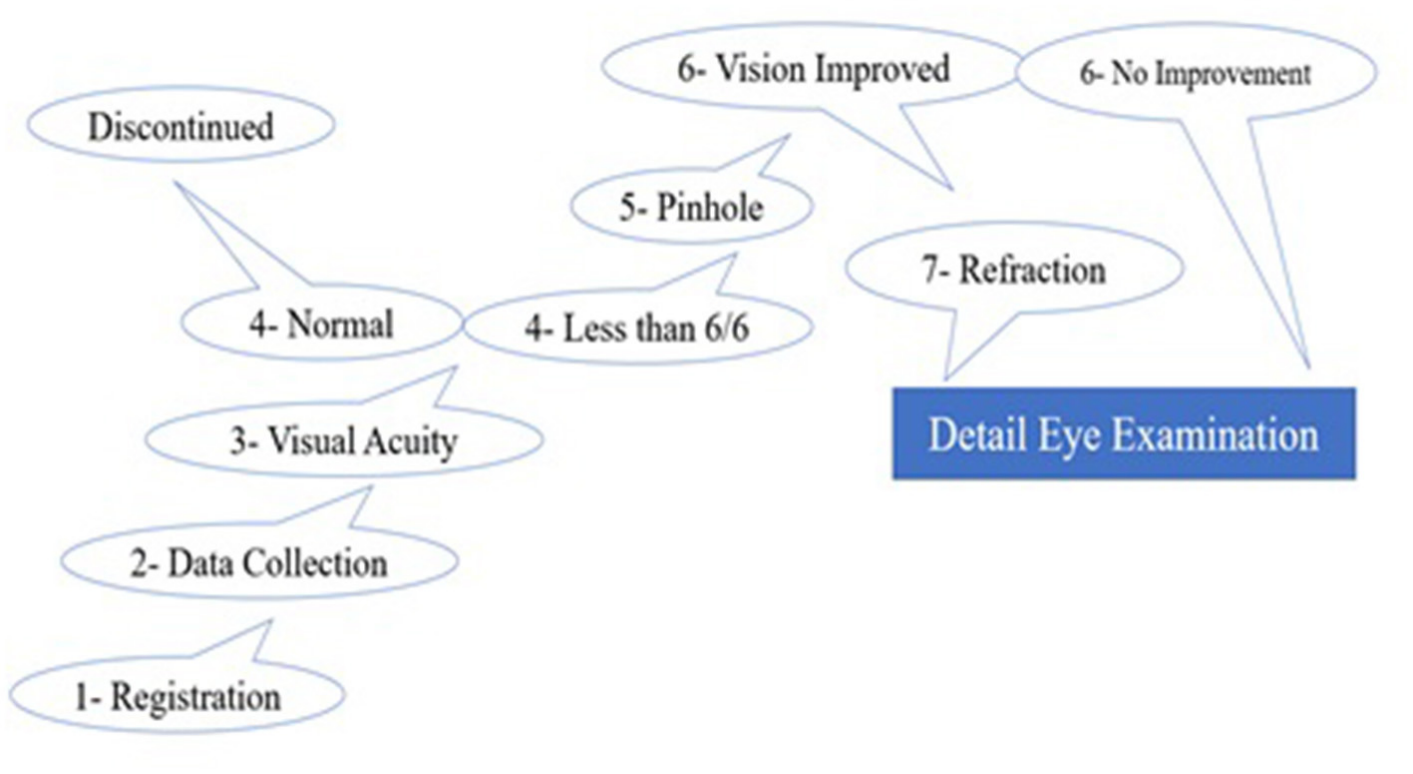
Schematic diagram of the screening protocols for measurement of refractive errors at school.

A structured questionnaire consisting of four sections was prepared for data collection. That was validated and pretested. The first component contained the basic information about the study subjects including the demographic profile, personal history, family history, and relevant medical history. The second part consisted of the questions regarding the educational status of the parents, learning activities of the participants after the school timing, and the academic performance of the study subjects. The third part of the questionnaire was developed to record the information about the ocular examination including visual acuity, pinhole examination, and refraction. The last part consisted of the final diagnosis and type of the refractive errors. The first two parts were filled by the trained research assistants whereas the third part was completed by the qualified optometrists and the last part of the questionnaire was filled by an ophthalmologist as shown in Figure 2.

### Screening for Refractive Errors

After the collection of basic information and relevant history, visual acuity was measured by using the illuminated Snellen chart separately for each eye placed at a distance of 6 m from the study participant. The students having a visual acuity of <6/6 in either eye were examined again by putting the pinhole in front of each eye for any improvement on the Snellen chart. The improvement of visual acuity by pinhole represents the presence of refractive errors. The students having a visual acuity of <6/6 in either eye underwent the objective and subjective refraction using an auto refractometer, retinoscopy, and subjective refraction. The ophthalmologist had the final examination of the student with a handheld slit lamp to see any ocular disease for inclusion or exclusion of the study subject.

### Data Collection About Academic Performance

The data were recorded on the questionnaire along with the academic performance of the students. The academic scores after intervention were assessed based on the next upcoming annual exam. The examination system is based on subjective and objective patterns evaluated by external boards of examinations on an annual basis. The annual examination, percentage score was assessed as academic performance in this study.

### Intervention

After 2–4 weeks, the researcher along with the professional team again visited the school and prepared spectacles of advised numbers that were distributed to the respective students. A training session for these students about the use of glasses was also conducted. The respective teachers were also trained to ensure the use of spectacles. Follow-up bi-monthly visits were also managed to ensure that students were wearing the spectacles.

### Statistical Analysis

The data were entered into version 21 of SPSS (Statistical Package for Social Sciences) and analyzed using statistical tests. The means were calculated for numeric variables, that is, age, academic score, etc. Categorical variables, for example, gender, family history, ocular examination, and refraction type were presented in the form of frequency and percentages. The mean academic score was taken before and after the intervention. Relevant statistical tests including chi-square, independent samples test, and ANOVA were applied. A *p-*value of ≤ 0.05 was considered statistically significant.

## Results

Out of the total 2,000 study participants, it was found that 412 (20.6%) were having refractive errors with 95% CI. The types of refractive errors were also investigated and myopia was found as the leading type 215 (52.2%) followed by astigmatism 136 (33%) and hypermetropia 61 (14.8%), respectively, among the 412 study participants having refractive errors. The prevalence was found higher among the public high schools 244 (59.2%) compared to private schools 168 (40.8%) while the area-based prevalence of refractive errors was found to be higher among the students of urban settings 255 (62%), while in rural, it was 157 (38%). These results were cross-tabulated and found statistically significant at a *p*-value < 0.01 regarding schools and localities and gender-based results.

With our methods, refractive errors were found prevalent among 412 (20.6%) out of the 2,000 study subjects. However, 159 (38.5%) of the participants having refractive errors were using corrected spectacles. They were excluded while 253 (61.5%) newly diagnosed candidates proceeded for intervention. The mean score of the results among the study participants before and after intervention were compared and are reported below.

Gender and area-based academic performance were compared and the results are presented in Table 1. There were no significant differences in the academic scores of urban vs. rural children, or between genders, before and after the intervention.

**Table 1:** Shows the gender, area, tuition, and refractive correction intervention effects on academics.

**Gender-based comparison of the academic performance**				
	**Gender**	**Mean**	**SD**	***p*** **-value**
Before intervention	Male (119)	57.54	16.48	0.220
	Female (134)	59.89	13.85	
After Intervention	Male (119)	63.88	15.98	0.204
	Female (134)	61.27	16.80	
**Area-based comparison of academic performance**				
	**Area**	**Mean**	**SD**	***p*** **-value**
Before Intervention	Rural	57.42	17.02	0.227
	Urban	59.76	13.66	
After Intervention	Rural	60.23	18.18	0.068
	Urban	64.04	15.00	
**Comparison of results (Public and Private) before and after intervention**				
	**Public**	**Private**	***p*** **-value**
Before Intervention	56.39 ± 13.24	63.53 ± 17.50	<0.001^*^
After Intervention	60.27 ± 14.94^†^^‡^	67.12 ± 18.48	<0.001^*^
**Comparison of effect of tuition on academic performance**				
	**Tuition**	**Public**	**Private**
Before Intervention	Yes	57.05 ± 13.03	62.39 ± 16.28
	No	55.68 ± 13.45	65.82 ± 21.27
After Intervention	Yes	62.01 ± 14.17^†^	68.36 ± 17.06^†^
	No	58.90 ± 14.58^†^	66.44 ± 20.36^†^

** *p-value significant at 0.05*.

† *Difference from before intervention was statistically significant*.

‡ *Statistically significant difference*.

The public and private sector results were also compared and it was found that, among the public sector, the average score of results before intervention was 56.39 ± 13.24 which increased to 60.27 ± 14.94 after the intervention. The average score of public schools at different times of study was found statistically significant at *p*-value < 0.05. Among the private sector, the before intervention average score was 63.53 ± 17.50, while after the intervention, it was 67.12 ± 18.48. Differences between the average score of private schools at different times of the study were statistically significant (*p*-value < 0.05) as shown in Table 1.

The mean score before the intervention was 57.05 ± 13.03 among the subjects of public schools with refractive errors who took tuition, while after the intervention, it was increased to 62.01 ± 14.17. Whereas, among students who did not take tuition, their mean score before the intervention was 55.68 ± 13.45 while after the intervention, it was improved to 58.90 ± 14.58. Among private school students, who took tuition, the mean score was 62.39 ± 16.28 before the intervention, while after the intervention, it was increased to 68.36 ± 17.06. Similarly, for students who did not take tuition, their mean score before intervention was 65.82 ± 21.27 which was increased to 66.44 ± 20.36, which was statistically significant as described in Table 1.

## Discussion

Refractive error has been discussed in the literature in detail. It is estimated that out of 2.3 billion people with refractive errors only 1.8 billion can have the access to affordable ophthalmic examination and their correction worldwide (14). So, accessibility and affordability are major problems but it is also worth mentioning that the management of these errors is considered among the most cost-effective interventions in health care (15).

The results of this study revealed that 20.6% of the study subjects were having refractive errors. Myopia was found as the leading type accounting for 52.2% of the refractive errors followed by astigmatism 33% and hypermetropia 14.8% among the 412 study participants having refractive errors. These findings are contradictory to the results of the previous studies in which the prevalence was 7% (16), 15.4% (17), 13.7% (18) 10.5% (19) 13.1% (20), and 11.6% (21), respectively. The results are similar regarding the type of refractive errors as myopia was the most common refractive error 61.9% (16) and 58.5% (19) in India. However, the findings of this study are comparable to a study of Muzaffarabad city in Azad Jammu and Kashmir concluding a prevalence of 19.6% (22) and 22% (23) in Saudi Arabia. However, the burden of the types of refractive errors revealed that the Libyan study is different from the findings of this study and conclude hyperopia 32.2% (23) and 53.2% (21) as the leading type. The prevalence of two similar studies in Ghana and Nepal concluded the 3.25 and 10.35% prevalence of refractive errors, respectively (21, 24). The low prevalence in the school of Ghana may be due to differences in methodology as this study's subjects were comparatively older children and cycloplegic refraction was not performed (21).

The findings of this study are similar to the results of some relevant research from Pakistan concluding a prevalence of 21.7 and 20.07% (9, 10). The results are against the findings of another similar study conducted among 1,644 schoolchildren of 5–15 years at Kohat, Pakistan, concluding the burden of refractive errors was 8.2%. However, this research was cross-sectional and a convenience-based sampling technique was opted (9).

The gender-based prevalence of refractive errors was analyzed among the present study subjects and it was found that the prevalence was high among the girls 238 (57.8%) as compared to boys 174 (42.2%). There was a statistically significant association between refractive error and the gender of the study participants (*p*-value < 0.001). These results are like the findings of another school-based research from Muzaffarabad which concluded a gender-based significant association (*p* = 0.001) and prevalence was higher among the female participants as compared to the male study subjects. However, this study reflects quite a high prevalence of 88.7% among the females and 11.3% prevalence among the male participants (22).

The results of the present research are in line with the findings of other similar studies concluding that the female students are having a higher prevalence of refractive errors as compared to the male students 7.86 (16), 15.3 (18), 52% (14), and 51.9% (14), respectively. The findings of the present study favor another study concluding a higher prevalence among the female participants (57.5%) as compared to the male subjects, 42.5% (19). However, these results were not statistically significant which contrasts with the findings of this study (19). The gender-based findings of the present study oppose the results of another cross-sectional Nigerian study conducted between the private and public schools among 1,197 subjects aged 8–15 years recruited through multistage random sampling. This research concluded that there was no statistically significant association between the prevalence of myopia among the male and female participants (25). These gender-based results are consistent with the findings of a relevant study reporting a high prevalence among the female study subjects (26). However, another study revealed a high magnitude among the male participants (27, 28).

The academic performance of the study participants was investigated and presented in the form of mean scores of the examination before and after the intervention. The cumulative groups mean score before intervention was found as 56.39 ± 13.24 and 63.53 ± 17.50 in public and private schools, while it was improved to 60.27 ± 14.94 and 67.12 ± 18.4 after the intervention, respectively; the subsequent, post-intervention examination results represented a clear improvement. The findings of after-intervention follow-ups are in clear contradiction to the findings of a study from Singapore which revealed that presenting visual acuity does not have a significant effect on the current or academic school performance after a year of the correction (29). The possible reason may be the differences in participant age between this and the Singapore study. However, the evidence of a randomized trial from rural China about the impact of spectacles on the academic performance of primary school children revealed an increased average score on the tests by the availability of glasses which corroborates the findings of this study (30). Similarly, another study also corresponds to the findings by concluding significant relations between the quality of academic performance and the presence of visual impairments (31). Another Chinese study about the effects of free glasses provision on the outcome of education concluded a statistically significant impact on academic performance which also favors the findings of this study (32).

## Conclusion

A significant impact was found in the mean score of results after intervention considering the adjustment of tuition and extra coaching after the school timing. Despite the presence of multiple public, private, and free eye care services in Lahore, diagnosis and management of refractive errors are still serious issues that directly influence academic achievement. The findings also reflect the state of affairs in the remote and deprived areas demanding an immediate response by the policymakers not only for the visual health but rather for improving the academic performance of the schoolchildren as well.

### Recommendations

**Figure 3:**
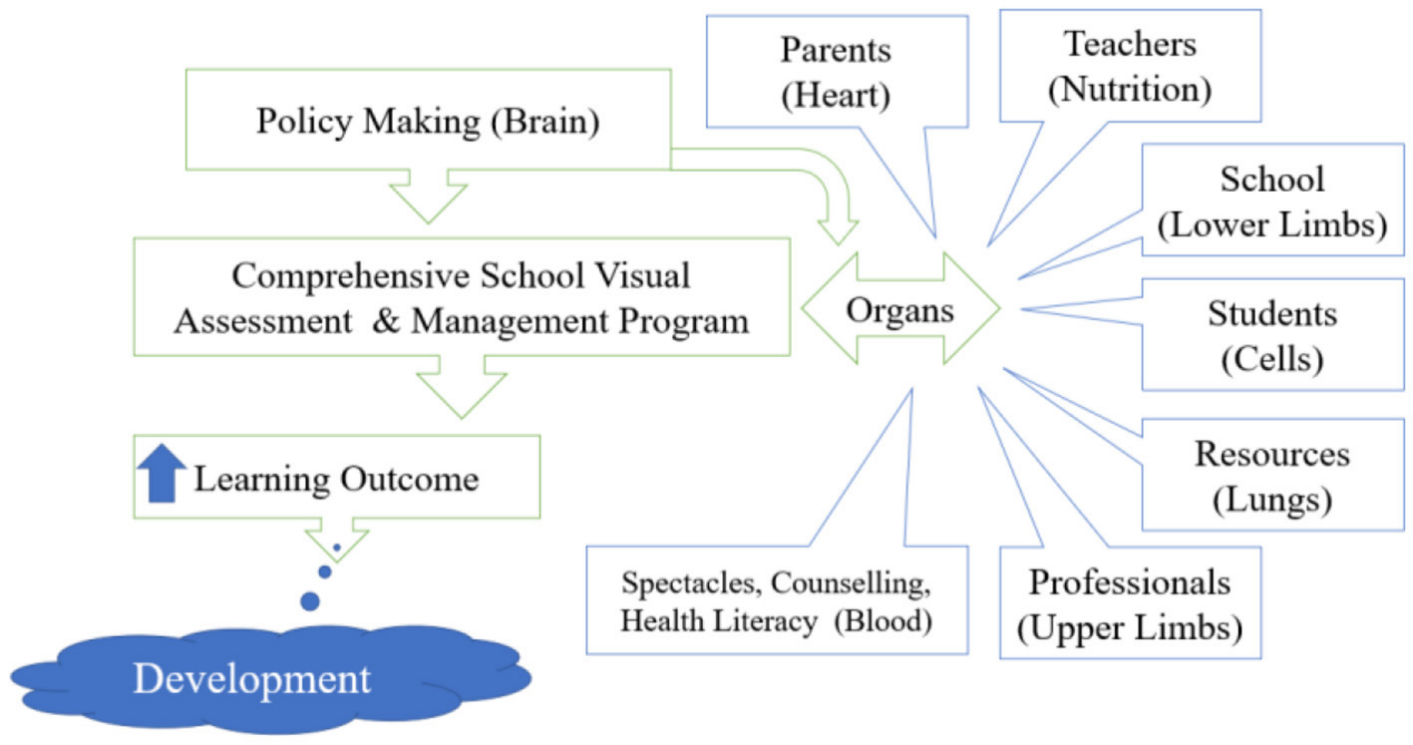
Proposed model for the Comprehensive School Eye Care Program (CSECP).

A comprehensive School Visual Assessment & Management Program (SVA&MP) with the provision of required spectacles is the need of the hour. A schematic diagram of SVA&MP is presented in Figure 3.

### Limitations

This study was conducted in Lahore having better educational and eye care facilities, which may limit the generalizability of the study. The study could reveal better results if a control group or prospective cohort design had opted. A multi-center study with an increased number of participants may result in a more precise and accurate outcome but the feasibility and resource management will be an uphill task.

Moreover, this research was conducted to study the impact of refractive errors on the academic performance of high school children in Lahore. Considering the sample size and issues regarding informed consent and cycloplegic effects, it was not feasible for us to make cycloplegic examinations in schools and this is a limitation of our study.

## Data Availability Statement

The datasets presented in this study can be found in online repositories. The names of the repository/repositories and accession number(s) can be found in the article/supplementary material.

## Ethics Statement

The studies involving human participants were reviewed and approved by Institutional Research Board of the University of Lahore vide letter reference number IRB-UOL-FAHS/00248A. Written informed consent to participate in this study was provided by the participants' legal guardian/next of kin.

## Author Contributions

ML: conception or design of the work and final approval of the version to be published. ML, IH, and SA: data collection. ML, MN, and SH: data analysis and interpretation. ML, MS, AA, SH, and MN: drafting the article. ML and SG: critical revision of the article. All authors contributed to the article and approved the submitted version.

## Conflict of Interest

The authors declare that the research was conducted in the absence of any commercial or financial relationships that could be construed as a potential conflict of interest.

## Publisher's Note

All claims expressed in this article are solely those of the authors and do not necessarily represent those of their affiliated organizations, or those of the publisher, the editors and the reviewers. Any product that may be evaluated in this article, or claim that may be made by its manufacturer, is not guaranteed or endorsed by the publisher.
